# Sexual behavior and drug consumption among young adults in a shantytown in Lima, Peru

**DOI:** 10.1186/1471-2458-9-23

**Published:** 2009-01-19

**Authors:** Juan A Gálvez-Buccollini, Suzanne DeLea, Phabiola M Herrera, Robert H Gilman, Valerie Paz-Soldan

**Affiliations:** 1 Asociación Benéfica PRISMA, Carlos Gonzales 251, Lima 32, Peru; 2 School of Medicine, University of New Mexico, Albuquerque, New Mexico 87131, New Mexico, USA; 3 Department of International Health, Bloomberg School of Public Health, Johns Hopkins University, 615 N Wolfe Street/W 5515, Baltimore, MD 21205, USA; 4 Tulane University School of Public Health and Tropical Medicine, 1440 Canal Street, Suite 2200, New Orleans, LA 70112, USA

## Abstract

**Background:**

Risky sexual behaviors of young adults have received increasing attention during the last decades. However, few studies have focused on the sexual behavior of young adults in shantytowns of Latin America. Specifically, studies on the association between sexual behaviors and other risk factors for sexually transmitted infections (STI) and HIV/AIDS transmission, such as the consumption of illicit drugs or alcohol are scarce in this specific context.

**Methods:**

The study participants were 393 men and 400 women between 18 and 30 years of age, from a shantytown in Lima, Peru. Data were obtained via survey: one section applied by a trained research assistant, and a self-reporting section. Logistic regression was used to estimate associations between use of any illicit drug, high-risk sexual behaviors and reported STI symptoms, adjusting for alcohol consumption level and various socio-demographic characteristics.

**Results:**

Among men, age of sexual debut was lower, number of lifetime sexual partners was higher, and there were higher risk types of sexual partners, compared to women. Though consistent condom use with casual partners was low in both groups, reported condom use at last intercourse was higher among men than women. Also, a lifetime history of illicit drug consumption decreased the probability of condom use at last sexual intercourse by half. Among men, the use of illicit drugs doubled the probability of intercourse with a casual partner during the last year and tripled the probability of reported STI symptoms.

**Conclusion:**

Drug consumption is associated with high-risk sexual behaviors and reported STI symptoms in a Lima shantytown after controlling for alcohol consumption level. Development of prevention programs for risky sexual behaviors, considering gender differences, is discussed.

## Background

In Peru, the HIV epidemic is still concentrated in high-risk populations, especially among men who have sex with men (MSM) [1]. The estimated HIV prevalence is 0.3% in the general population, 1% among sex workers, and between 14% to 22% among MSM [2,3]. Although HIV incidence in Peru has remained constant during the last decade, the incidence among heterosexual women has increased. This is believed to be due to a bridge population of men who engage in sexual relations with both high and low-risk populations [4].

Risky sexual behaviors of young adults have received increasing attention in the last two decades [5-9]. It is believed that more than two million adolescents become infected with HIV worldwide every year. The most common form of transmission in this group is sexual intercourse. Within at risk populations, young adults are at highest risk of acquiring sexually transmitted infections (STIs) and HIV because most are sexually active, single, have multiple partners since they are not in established monogamous relationships, engage in intercourse with sex workers, and frequently mix sex with alcohol or drugs [10-13]. Previous research conducted mainly in developed countries has found that high-risk sexual behaviors are associated with an individual's history of drug and alcohol consumption [12-14]. Therefore, it is necessary to control for alcohol consumption levels in order to establish the independent effect of illicit drug use on sexual behavior. Moreover, it is likely that varying cultural and environmental factors related to young adults' sexual practices (such as how acceptable it is for a young man to be initiated into sex by having sex with a sex worker or having casual partners) and drug use (such as the availability or popularity of different types of drugs) influence this association in different ways in different regions of the world [15-18].

Drug consumption and its health consequences are an increasing problem in Peru. Between the year 2003 and 2005, the national survey on the use of drugs registered a 3% increase in the number of people who admitted to consuming an illegal drug at least once in their lifetime, from 11% to 14% [19]. Although Peru is one of the main producers of coca leaves in the world, marijuana (11.9%) remains the most consumed drug over a person's lifetime, followed by coca paste (3.7%) and cocaine (3.4%) [19].

Several studies on sexual behavior among young adults in urban areas have been conducted in Latin America [6,20-22]. They have found, for example, that 80% of young adults between 18 and 29 years of age in Peru have had intercourse, and that of this group, 30% of young men report having had intercourse with a sex worker [20]. However, few studies have focused on the sexual behavior of this population in shantytowns of Latin America [18]. Thus, information about attitudes and behaviors related to sexual health in this population is limited. Specifically, studies on the association between sexual behaviors and other risk factors for STI and HIV transmission, such as the consumption of illicit drugs or alcohol, are scarce in this specific context.

This study describes the reported sexual behaviors of young adults in a shantytown in Lima, Peru, by gender, and examines the association between drug consumption and sexual behaviors. We specifically examined the association between drug consumption and use of a condom during last sexual intercourse, both unadjusted and adjusting for alcohol consumption level and other socio-demographic variables. Additionally, among men, we estimated the association between drug consumption and the following variables: 1) intercourse with a casual partner in the past year, 2) intercourse with high-risk sexual partners in the past three months, and 3) ever reporting STI symptoms. Examining the effect of alcohol and illicit drug use jointly would reveal the relative effects of alcohol and illicit drug use on the occurrence of risky sexual behaviors and reported STI symptoms.

## Methods

### Study site

This cross-sectional study took place in Las Pampas de San Juan de Miraflores (PSJM), a shantytown with an estimated population of 40,000 located approximately 15 kilometers south of downtown Lima, Peru. As in many other shantytowns in Peru, PSJM was formed by residents who settled and later claimed unused land on the outskirts of Lima. Physically, this environment consists of steep and rocky desert hills with dusty soil, no natural vegetation, and less than one inch of annual rainfall. Most households have electricity, but only about half have water or sewage connections. Underemployment and poverty are high in PSJM. The estimated annual median income in PSJM was $2100 in 2000, compared to $7600 for the rest of the country [23,24]. Only about 25% are stably employed. This area was settled over 20 years ago, predominantly by individuals who emigrated from the Peruvian highlands. However, most young people were born and raised in PSJM and thus are fluent in Spanish despite their parents' region of origin. Since 1986, the community of PSJM has been under health surveillance for various health research projects by physicians, public health care workers, nurses and social workers from the Peruvian non-governmental organization A.B. PRISMA.

### Sample

All PSJM residents were registered in a census completed by A. B. PRISMA for previous epidemiological studies [25-27]. Young adult residents of the shantytown between the ages of 18 and 30 were randomly selected from this census and asked to participate in our study. A random sample of potential participants from the census list was generated using the computer program Visual Fox Pro 9.0. A total of 968 subjects (508 men and 460 women) were contacted to participate in our study by trained personnel. Of those contacted, 16 declined to participate (11 men and 5 women), and 152 were unavailable for interviews (97 men and 55 women). Seven male participants were later excluded from the data analysis due to incomplete questionnaires. Hence, the total sample size was 793 subjects (81.9% response rate). Information from subjects who did not consent to participating or were unavailable to be interviewed was not collected.

### Design

Trained personnel with medical research experience in this community visited each eligible participant's home. Study objectives, risks and benefits were explained to the eligible participants at that time, stressing that participation was voluntary and could be discontinued at any time. Individuals who provided written consent were enrolled. If an eligible participant was not home at the first visit, new attempts to reach those individuals at their homes were made up to three times per individual. All interviews were conducted in Spanish. Though there may be some older adults whose native language is not Spanish, all of the young residents in this community are Spanish-speaking or bilingual. Interviewers completed a month long training course that included role-playing and mock interviews in order to increase the instruments' reliability. Our cross sectional study had two parts: an interview administered by the trained personnel that lasted approximately 25 minutes about the interviewees' sociodemographic background, and alcohol and drug use; and a self-administered questionnaire about sexual behavior that lasted approximately 15 minutes.

This study was reviewed and approved by the local non-governmental organization A.B. PRISMA Institutional Review Board (IRB) FWA# 00001219 for protection of human subjects in research. IRB approval was also obtained from Tulane University School of Public Health and Tropical Medicine for analysis of these data.

### Variables

Participants who responded affirmatively to "Have you ever had sexual intercourse?" were asked about condom use at last sexual intercourse, and the number of sex partners they had: 1) in the last three months, 2) in the last year prior to assessment, and 3) in their lifetime. We also asked participants whether in the past year they had engaged in sex with potentially high-risk sexual partners, such as casual partners, same sex partners and/or sex workers, and whether they had ever had an STI. In addition, individuals were asked about having ever had STI symptoms, specifically urethral or vaginal discharge and ulcers or anogenital lesions.

The risky sexual behaviors of interest examined in this study were: 1) use of a condom during last sexual intercourse, 2) intercourse with a casual partner in the past year, and 3) intercourse with high-risk sexual partners in the past three months. Our final dependent variable of interest was whether the participant reported having ever had STI symptoms.

A stable sexual partner was defined as a partner to "whom you feel committed to above anyone else, such as your husband or boyfriend". Casual partner was defined as "someone you have had sex with other than your stable partner or sex for pay". It could include casual acquaintances, one-night stands or someone that they had just met in a bar or disco [28].

The main independent variable of interest was lifetime history of illicit drug use. We asked specifically about use of marijuana, coca paste and cocaine. Intravenous drug use was not included as a drug of interest in this study because it is not a commonly used type of drug in Peru [29].

Regarding alcohol consumption level, which we controlled for, a heavy episodic drinker (HED) was defined as a subject who consumed at least 5 alcoholic drinks in a row at least once per month every month for the last 12 months, as in Slutske (2005) [30].

Standard demographic variables adjusted for included sex, age (continuous), marital status (single/divorced vs. married/cohabitant) and educational level (high school or less vs. more than high school).

### Data analysis

Logistic regression techniques were used to estimate association between ever use of illicit drugs (main covariate of interest), and risky sexual behaviors and reported STI symptoms, controlling for socio-demographic variables in the first model for each of the four outcomes of interest, as well as alcohol consumption level in the second model for each of the four outcomes of interest. Although sociodemographic variables such as education and employment were not associated with our targeted risky sexual behaviors nor illicit drug use, we controlled for them in the analysis [31-33]. The results of these analyses are presented in the form of adjusted odds ratios, using 95% confidence intervals; p-values are presented as an aid to interpretation. All analyses were done using STATA 8.0.

Before conducting the multivariate analysis, we also performed an exploratory analysis to identify potential effect modifiers. We tested the interaction between illicit drugs and sex, and heavy episodic drinking and sex. None of the interaction terms were significant (p > 0.2).

## Results

### Sample characteristics

The demographic characteristics of young adults are outlined in Table 1. By design, the sample consisted of similar numbers of men and women, of similar ages. However, there were differences in marital status, education, and work status (see Table 1 for percentages). It is interesting to note that despite women having more education than men, the proportion of unemployment was higher among women. More women had at least one child compared to men, but the rate of unplanned pregnancies was similar among them. With regards to condom availability, more women than men felt it would be difficult to get them.

**Table 1:** Sociodemographic, behavioral, and reproductive characteristics of the study sample by gender (n = 793)

	**Men** **n = 393**		**Women** **n = 400**		p*
	**%**	n	**%**	n	

Age mean (SD)	22.3	(3.2)	22.4	(3.3)	0.777
					
**Marital Status**					
Single	66.0	264	84.0	330	< 0.001
Married/Cohabitant	16.0	63	34.0	136	
					
**Education Level**					
High school or less	69.5	273	54.5	218	< 0.001
More than high school	30.5	120	45.5	182	
					
**Occupation**					
Employed	32.3	127	24.5	98	< 0.001
Under-employed	30.8	121	12.8	51	
Unemployed	16.3	64	36.0	144	
Student	20.6	81	26.8	107	
					
**Have children**	16.3	64	38.0	152	< 0.001
Have had unplanned pregnancy	54.7	35	61.2	93	0.375
					
**Ever had STI**	1.0	4	1.3	5	0.758
					
**Reported lifetime STI symptoms**					
Genital ulcers or sores	3.6	14	3.3	13	0.808
Urethritis	3.3	13	-	-	-
Vaginal discharge	-	-	26.8	107	-
					
**Consider it difficult to get condoms**	10.2	40	46.8	187	< 0.001
					
**Ever drug use**					
Marijuana	21.9	86	1.0	4	< 0.001
Coca Paste	5.9	23	0.3	1	< 0.001
Cocaine	4.8	19	0.3	1	< 0.001
					
**Heavy episodic drinking**	31.3	123	2.8	11	< 0.001
					
**Ever use of any illicit drug**	24.2	95	1.5	6	< 0.001

n: Number of participants

*chi square/kruskal-wallis

Few participants reported having had an STI (see Table 1). However, when we asked about specific STI symptoms (vaginal or urethral discharge, ulcers or anogenital lesions), the number was higher. Among women, 27% reported having had vaginal discharge, though we recognize that a vaginal discharge could be unrelated to STIs.

There were large differences in the heavy episodic drinking (31.3% vs. 2.8%) and in the lifetime use of illicit drugs (24% vs. 2%) between men and women. Marijuana was the most commonly used drug for both sexes (see Table 1).

### Sexual behavior

Most men (80%) reported having ever had sex, compared to 65% of women. The age of sexual initiation was two years younger for men than for women (16.9 ± 2.4 vs. 18.4 ± 2.5). As expected, more men reported having had casual sex partners than women (23.4% vs. 1.5%, p < 0.001). Only men reported sex with commercial sex workers or same sex partners. Additionally, 12% of men had sex with a casual partner, sex worker or same sex partner under the influence of alcohol during the last three months, compared to less than 1% among women. Also, seven men had sex with a casual partner, sex worker or another man while under the influence of an illicit drug during the last three months (Table 2).

**Table 2:** Sexual behaviors of 18 to 30 year olds in PSJM sample (n = 574)

	**Men**		**Women**		p*
	%	n	%	n	

**Ever had sex**	79.4	312	65.5	262	< 0.001
**Age of sexual debut **mean (SD)	16.9 (2.4)		18.4 (2.5)		< 0.001
					
**Ever had sex with**					
Casual partner	27.2	85	1.9	5	< 0.001
Sex worker	14.1	44	-	-	-
Same sex person	1.6	5	-	-	-
					
**Last year had sex with**					
Casual partner	23.4	73	1.5	4	< 0.001
Sex worker	6.7	21	-	-	-
Same sex person	1.3	4	-	-	-
					
**Sex under alcohol influence with casual partners, FSW or SSP**^£^	12.2	38	0.8	2	< 0.001
**Sex under drug influence with casual partners, FSW or SSP**	2.2	7	-	-	-
					
**Number of sexual partners (reporting mean, SD)**					
Last 3 months	1.5 (2.0)		1.0 (0.1)		< 0.001
Last year	2.2 (3.1)		1.1 (0.4)		< 0.001
Lifetime	5.2 (12.1)		1.5 (1.0)		< 0.001
					
**Number of casual partners last year**	3.2 (5.2)		1.3 (0.5)		0.076
**Number of FSW last year**	2.0 (1.5)		-		-
					
**Unprotected sex (non-condom use)**					
At last sexual intercourse	51.3	160	70.2	184	< 0.001
At last intercourse with casual partner	27.1	23	40.0	2	0.530
At last intercourse with FSW	4.6	2	-	-	-
					
**Always condom use**					
Casual partner	44.7	38	40.0	2	0.837
Sex worker	84.1	37	-	-	-

n: Number of participants

*chi square/kruskal-wallis

^£ ^SSP: same sex partner

Men had more sexual partners than women in all the assessed time periods: men had a higher number of sex partners in the past year (2.2 ± 3.1 vs. 1.1 ± 0.4) and lifetime number of sexual partners (5.2 ± 12.1 vs. 1.5 ± 1.0) than women. However, as is reported, the standard deviations for these estimates are high, so these results have to be carefully interpreted. Among those who reported having casual partners during the last year, men also had a greater number of partners than women (3.2 ± 5.2 vs. 1.3 ± 0.5, p = 0.076).

More men reported condom use at last intercourse than women (48.7% vs. 29.8%, p < 0.001). Differences in condom use between the genders remained significantly different during the last sexual encounter when we analyzed only participants who were single (condom use: men 56%, women 43%, p = 0.021). Reported condom use was also higher among men than women during the last sexual intercourse with a casual partner, but this difference was not statistically significant. However, consistent condom use with casual partners was low in both groups (45% among men and 40% among women, p = 0.837). Among men, only 84% used condoms consistently during sexual intercourse with female sex workers. More than 95% of men reported using condoms during their last sexual intercourse with a female sex worker.

### Illicit drug use, heavy episodic drinking and risky sexual behaviors

We proceeded to estimate the association between condom use at last intercourse and lifetime history of illicit drug use, adjusting for sociodemographic variables (gender, age, marital status, education and work status). We found that being male, single and having ever used any illicit drug (adjusted Odds Ratio [aOR] = 0.5, 95% CI = 0.3–0.8, p = 0.006) were all associated with condom use at last intercourse (Table 3: model 1a). The effect of adding HED to the adjusted model was not significant and there were no significant shifts in any of the estimates (Table 3: model 2a).

**Table 3:** Adjusted association between illicit drug use and condom use among men and women between 18 to 30 years in PSJM sample (n = 574).

	**Condom use at last sexual intercourse**			
	**Model 1a**		**Model 2a**	

	**aOR 95%CI**	**p**	**aOR** **95% CI**	**p**

**Heavy episodic drinking**^€^	-	-	1.2	0.469
			(0.7–2.0)	
Ever illicit drug use*	0.5	0.006	0.5	0.005
	(0.3–0.8)		(0.3–0.8)	
**Sex**^§^	1.9	0.004	1.8	0.008
	(1.2–2.8)		(1.2–2.7)	
**Age **^Ψ^	1.0	0.901	1.0	0.900
	(0.9–1.1)		(0.9–1.1)	
**Education **^£^	1.2	0.305	1.2	0.283
	(0.8–1.8)		(0.8–1.8)	
**Employment**^¥^	0.9	0.638	0.9	0.605
	(0.6–1.4)		(0.6–1.4)	
**Marital status**^∞^	4.4	<0.001	4.4	< 0.001
	(2.8–7.0)		(2.8–7.0)	

^€ ^Heavy episodic drinking: yes vs. no (ref)

* Ever use illicit drug: yes vs. no (ref)

^§ ^Men vs. women (ref)

Ψ Age: continuous

£ Educational level: High school or less vs. More than high school (ref)

¥ Employment: unemployed vs. employed or student (ref)

∞ Marital status: single vs. married or cohabitant (ref)

All the remaining multivariate analyses in this paper were only conducted on men because there were few, if any, women who reported ever using illicit drugs (n = 6), casual sex (n = 4), and sex with a casual partner, sex worker or a same sex partner under the influence of alcohol during the last three months (n = 2).

Hence, among men, we estimated the association between having had casual sex in the past year and lifetime history of illicit drug use, again adjusting by sociodemographic variables. We found that lifetime history of drug use, being single and being younger were associated with having had casual sex in the past year (Table 4: model 1b). After HED was added to the adjusted model, it was also associated with men having had casual sex in the past year, and there was a slight decrease in the association between lifetime history of drug use and having had casual sex in the past year after HED was added (Table 4: model 2b).

**Table 4:** Adjusted association between illicit drug use, and reported risky sexual behaviors and STI symptoms among men between 18 to 30 years in PSJM sample (n = 312).

	**Intercourse with a** **casual partner during ** **the last year**				**Sex with a casual partner, ** **sex worker or MSM under ** **the influence of alcohol** **during the last 3 months**				**Ever had any STI symptom**			
	**Model 1b**		**Model 2b**		**Model 1c**		**Model 2c**		**Model 1d**		**Model 2d**	

	**aOR 95% CI**	**p**	**aOR** **95% CI**	**p**	**aOR** **95% CI**	**p**	**aOR** **95% CI**	**p**	**aOR** **95% CI**	**p**	**aOR** **95% CI**	**p**

**Heavy episodic drinking**^€^	-	-	2.0	0.019	-	-	3.1	0.004	-	-	1.0	0.945
			(1.1–3.7)				(1.4–6.8)				(0.4–2.7)	
Ever illicit drug use*	3.2	< 0.001	2.5	0.002	2.5	0.012	1.8	0.147	3.4	0.007	3.3	0.014
	(1.8–5.6)		(1.4–4.7)		(1.2–5.2)		(0.8–3.8)		(1.4–8.1)		(1.3–8.6)	
**Age **^Ψ^	0.9	0.042	0.9	0.044	0.9	0.117	0.9	0.158	1.0	0.546	1.0	0.545
	(0.8–0.9)		(0.8–0.9)		(0.8–1.0)		(0.8–1.0)		(0.9–1.2)		(0.9–1.2)	
**Education **^£^	1.2	0.564	1.2	0.496	1.6	0.209	1.7	0.171	1.4	0.468	1.4	0.467
	(0.6–2.2)		(0.7–2.3)		(0.8–3.6)		(0.8–3.9)		(0.5–3.8)		(0.5–3.9)	
**Employment**^¥^	1.3	0.411	1.2	0.604	3.0	0.007	2.7	0.022	1.9	0.273	1.9	0.274
	(0.7–2.8)		(0.6–2.5)		(1.4–6.8)		(1.2–6.2)		(0.6–5.7)		(0.6–5.7)	
**Marital status ∞**	4.6	0.006	4.5	0.007	3.7	0.088	3.4	0.108	0.4	0.070	0.4	0.070
	(1.5–13.7)		(1.5–13.6)		(0.8–16.4)		(0.8–15.4)		(0.1–1.1)		(0.1–1.1)	

^€ ^Heavy episodic drinking: yes vs. no (ref)

* Ever use illicit drug: yes vs. no (ref)

Ψ Age: continuous

£ Educational level: High school or less vs. College (ref)

¥ Employment: unemployed vs. employed or student (ref)

∞ Marital status: single vs. married or cohabitant (ref)

In the model adjusting only for sociodemographic characteristics, we found that having ever used illicit drugs and being unemployed were associated with ever having had sex with a casual partner, sex worker or another male under the influence of alcohol during the last three months (Table 3: model 1c). After HED was added to the adjusted model, it was also associated with this risky behavior (Table 3: model 2c), but the association between lifetime history of drug use and this outcome was no longer significant in this model.

Finally, again only for men, we estimated the association between ever reporting any STI symptom and having ever used any illicit drug. In the model adjusting for sociodemographic variables, we found that lifetime history of illicit drug use was significantly associated with reporting STI symptoms (aOR = 3.4, 95% CI = 1.4–8.1, p = 0.007) (Table 3: model 1d). When HED was added to the adjusted model, HED was not found to be significantly associated with ever reporting STI symptoms, and there were no significant shifts in any of the other estimates (Table 3: model 2d).

## Discussion and Conclusion

In this study, the consumption of illicit drugs is associated with some high-risk sexual behaviors and with reported STI symptoms in this population of young adults living in a shantytown in Peru, even after controlling for alcohol consumption. As expected, men reported more risky sexual behaviors than women, but their reported condom use at last sex was also higher than among women. However, consistent condom use was low in both groups, especially with casual partners, and it is possible that because men report more casual partners than women, that their higher reported condom use relates to condom use with this type of sexual partner.

An important limitation to the interpretation of our results is that the study was retrospective, hence some information may have been forgotten by respondents. Second, there may be some social desirability bias in the responses about sexual behavior. The large discrepancy between data reported by men and women is consistent with the literature: one in which men exaggerate the report of certain sexual behaviors, while women underreport those same sexual behaviors [21,34]. Though several strategies were used to reduce this bias (training of interviewers, previous evaluation of the instrument, self-reporting, and privacy while survey was administered), it may not have been sufficient. However, some studies suggest that using self-reporting, which was the case in this study for sexual activity, is reliable for recording sexual behavior [35]. Third, alcohol and illicit drug use questions may be subject to social desirability bias as well: we might expect that some participants may have been more likely to under-report them. However, our rates of illicit drug use are higher than those found in the national survey on the use of drugs in 2005 [19], which increases our confidence in our results. Fourth, though use of self-reporting is not appropriate with low literacy populations, we do not believe this was a significant problem amongst our population because: 1) based on the last Demographic and Health Survey in Peru, illiteracy levels in Lima for this age group are less than 0.5% [36], and 2) participants had to read the consent form and interviewers had to verify that they had understood it before enrolling them. Fifth, the cross-sectional and retrospective design of our study does not allow us to establish a causal association between the consumption of illicit drugs and risky sexual behaviors. Also, having ever consumed illicit drugs might be associated with other characteristics of the participants, such as antisocial personality traits, sensation seeking behavior or mental disorders, which might better explain this association [12,37-39]. Also, some participants (17%) did not consent to participating or were unavailable to be interviewed, and though we do not know why they did not participate, they could be a source of bias for our results. Finally, there were few participants who reported risky sexual behaviors, being heavy episodic drinkers and who had consumed drugs at least once in their lifetime, especially among women, making it difficult to analyze these data with statistical significance due to insufficient power.

Nevertheless, our results are interesting because there are few studies that describe sexual behavior and drug consumption in shantytowns in Latin America, especially after adjusting for HED. The lifetime prevalence of illicit drug use was higher in our study than that reported for the same age group in the national survey on the use of drugs in 2005 (24% vs. 19%) [19]. It is possible that these differences are due to varying sociodemographic characteristics, including the low socioeconomic status of people living in the shantytown [39]. This study also found that HED and drug consumption were independently associated with some sexual behaviors such as having had high-risk sexual encounters during the last year. However, not using a condom during the last sexual encounter and reporting STI symptoms, direct consequence of having risky sexual behaviors, were only associated with having ever used illicit drugs. These findings are consistent with other studies [37,40]. Although only HED was associated with having had risky sexual partners under the influence of alcohol in the adjusted model (vs. HED and ever use of any illicit drug), it is possible that our study did not have enough power to disentangle this difference. It has been suggested that alcohol and drug use interferes with the process of reasoning and decision-making, increasing the chance of high-risk sexual behaviors and consequently, STIs [22]. However, considering that we cannot establish any temporal or causal relationship between lifetime history of alcohol or illicit drug use and high-risk sexual behaviors, it is possible that those factors are maladaptive behaviors that share a common origin [12,39]. It is clear, however, that efforts to prevent STI and HIV should focus on those who ever used any illicit drug.

With regards to gender differences in sexual behaviors, most of the results found in this study were quite similar to those of other studies in Peru [20]. However, the proportion of women that had casual sexual encounters during their lifetime (1.5%) in our study was low compared to the 5% of women that reported their last sexual partner as a casual partner in the National Survey of STI and HIV Prevalence in young adults in Peru [20]. Though it is unclear why our numbers varied from the other survey, it may be because most of the residents in this shantytown originally came from the Peruvian highlands, with a more traditional conservative culture, especially related to sexual behavior: expression of one's sexuality in the highlands is less observed than in the coast or rainforest of Peru [6,41]. This fact could also explain the lower proportion of men reporting ever having had sex with female sex workers than that reported in the National Survey (14% vs. 29%) [20].

This study finds that the use of illicit drugs doubled the probability of intercourse with a casual partner in the past year and tripled the probability of reported STI symptoms, indicating that this sub-group represents an important target population for HIV/STI prevention. However, in order to develop programmatic recommendations, more research is needed to examine this association with a larger sample for more power in the analysis. Specifically, it would be important to determine whether the association between illicit drug use and high risk sexual activities occurred during the same time period of a person's life (i.e., the drug use and high risk sexual behavior were concurrent vs. one behavior leading to the other), to examine whether the association between illicit drug use and risky sexual behaviors is stronger for certain drugs, and to explore the mechanism implicated in this association. Alternatively, it would be essential to determine if there are inherent characteristics in certain individuals that may lead to all types of higher risk behaviors – from illicit drug use to high risk sexual activities.

Finally, our findings have programmatic implications for HIV/STI prevention efforts in poor dense urban areas, such as in this shantytown. First, gender specific culturally tailored prevention efforts need to target different behaviors for men and women. Specifically, condom use among women needs to be highly stressed. The fact that more men report using condoms than women may be due to their higher risk sexual behaviors. However, women are contracting STIs and HIV from their stable partners, so they need to understand the importance of using condoms with any type of partner, whether women perceive their male partners to be faithful or "risky". Thus, given gender differences in sexual behavior found in this study, interventions for women need to be oriented towards improving their perceptions of personal risk, and learning about various protective behaviors. Moreover, due to "machismo", it is important for programs to teach women negotiating skills on using condoms with their partners: women may be aware of the importance of using condoms, but are obviously not using them, and it may be due to a discomfort or inability to request condom use from their partners. Related to the machismo and condom use, it is possible that one reason that 50% of women in our study reported difficulty getting condoms where they live is due to embarrassment about being sexually active: condoms need to be made available in locations where women can access them anonymously. Among males, interventions may need to be focused on decreasing the trend for engaging in risky sexual behaviors, which may have to focus on changing cultural norms, and improving their perception of personal costs (i.e., getting an STI) associated with these behaviors. Although "machismo" is perceived, in many settings, as something negative because it implies women's subjugation, in some locations it is also associated with being a good provider and being protective with your partner and family [42]. Hence, using this "machismo" concept, preventive efforts could aim at the message that men are protecting their partners by using condoms. Last, but perhaps most importantly, interventions should address the existing double standard in which risky sexual behaviors and other risk behaviors, such as heavy drinking or drug use, are culturally accepted among men and discouraged among women, and instead promoting responsible and safe sexual lives for both.

## Competing interests

The authors declare that they have no competing interests.

## Authors' contributions

All five authors participated in the design of the study. JAGB was the main person responsible for the data collection. JAGB conducted all the statistical analysis in this article. JAGB and VPS were the main ones responsible for the writing of the article. All five authors participated in discussions of the findings that led to this article, interpretation of findings, and editing the manuscript. All authors read and approved the final content of the manuscript.

## Pre-publication history

The pre-publication history for this paper can be accessed here:



## References

[B1] Bautista CT, Sanchez JL, Montano SM, Laguna-Torres VA, Lama JR, Sanchez JL, Kusunoki L, Manrique H, Acosta J, Montoya O (2004). Seroprevalence of and risk factors for HIV-1 infection among South American men who have sex with men. Sex Transm Infect.

[B2] Sanchez J, Lama JR, Kusunoki L, Manrique H, Goicochea P, Lucchetti A, Rouillon M, Pun M, Suarez L, Montano S (2007). HIV-1, sexually transmitted infections, and sexual behavior trends among men who have sex with men in Lima, Peru. J Acquir Immune Defic Syndr.

[B3] Lama JR, Lucchetti A, Suarez L, Laguna-Torres VA, Guanira JV, Pun M, Montano SM, Celum CL, Carr JK, Sanchez J (2006). Association of herpes simplex virus type 2 infection and syphilis with human immunodeficiency virus infection among men who have sex with men in Peru. J Infect Dis.

[B4] Caceres CF, Mendoza W (2004). Monitoring trends in sexual behaviour and HIV/STIs in Peru: are available data sufficient?. Sex Transm Infect.

[B5] Hahm HC, Lee J, Ozonoff A, Amodeo M (2007). Predictors of STDs among Asian and Pacific Islander young adults. Perspect Sex Reprod Health.

[B6] Magnani RJ, Seiber EE, Gutierrez EZ, Vereau D (2001). Correlates of sexual activity and condom use among secondary-school students in urban Peru. Stud Fam Plann.

[B7] Morrison DM, Gillmore MR, Hoppe MJ, Gaylord J, Leigh BC, Rainey D (2003). Adolescent drinking and sex: findings from a daily diary study. Perspect Sex Reprod Health.

[B8] Gerressu M, Stephenson JM (2008). Sexual behaviour in young people. Curr Opin Infect Dis.

[B9] Cavazos-Rehg PA, Spitznagel EL, Bucholz KK, Norberg K, Reich W, Nurnberger J, Hesselbrock V, Kramer J, Kuperman S, Bierut LJ (2007). The relationship between alcohol problems and dependence, conduct problems and diagnosis, and number of sex partners in a sample of young adults. Alcohol Clin Exp Res.

[B10] Alemu H, Mariam DH, Belay KA, Davey G (2007). Factors predisposing out-of-school youths to HIV/AIDS-related risky sexual behaviour in northwest Ethiopia. J Health Popul Nutr.

[B11] Staton M, Leukefeld C, Logan TK, Zimmerman R, Lynam D, Milich R, Martin C, McClanahan K, Clayton R (1999). Risky sex behavior and substance use among young adults. Health Soc Work.

[B12] Tubman JG, Gil AG, Wagner EF, Artigues H (2003). Patterns of sexual risk behaviors and psychiatric disorders in a community sample of young adults. J Behav Med.

[B13] Sly DF, Quadagno D, Harrison DF, Eberstein I, Riehman K (1997). The association between substance use, condom use and sexual risk among low-income women. Fam Plann Perspect.

[B14] O'Hare T (2005). Risky sex and drinking contexts in freshman first offenders. Addict Behav.

[B15] Nazar-Beutelspacher A, Tapia-Conyer R, Villa-Romero A, Leon-Alvarez G, Medina-Mora ME, Salvatierra-Izaba B (1994). Factors related to drug use by adolescents in urban areas of Mexico. Salud Publica Mex.

[B16] Becher JC, Garcia JG, Kaplan DW, Gil AR, Li J, Main D, Herrera JA, Arias L, Bromet A (1999). Reproductive health risk behavior survey of Colombian high school students. J Adolesc Health.

[B17] Peres CA, Rutherford G, Borges G, Galano E, Hudes ES, Hearst N (2008). Family structure and adolescent sexual behavior in a poor area of Sao Paulo, Brazil. J Adolesc Health.

[B18] Castro R, Orozco E, Eroza E, Manca MC, Hernandez JJ, Aggleton P (1998). AIDS-related illness trajectories in Mexico: findings from a qualitative study in two marginalized communities. AIDS Care.

[B19] Zavaleta A, Castro de La Mata R (2002). Epidemiologia de drogas en la poblacion urbana peruana 2005.

[B20] Cotrina A, Garcia P, Carcamo C (2008). Encuesta Nacional de Prevalencia de ITS y VIH en Poblacion General. Presentacion de resultados proyecto PREVEN: 2008.

[B21] Sanchez J, Gotuzzo E, Escamilla J, Carrillo C, Phillips IA, Barrios C, Stamm WE, Ashley RL, Kreiss JK, Holmes KK (1996). Gender differences in sexual practices and sexually transmitted infections among adults in Lima, Peru. Am J Public Health.

[B22] Brook DW, Brook JS, Pahl T, Montoya I (2002). The longitudinal relationship between drug use and risky sexual behaviors among colombian adolescents. Arch Pediatr Adolesc Med.

[B23] Reyes JOL (2006). Encuesta demográfica y de salud familiar.

[B24] Saito M, Bautista CT, Gilman RH, Bowering A, Levy MZ, Evans CA (2004). The value of counting BCG scars for interpretation of tuberculin skin tests in a tuberculosis hyperendemic shantytown, Peru. Int J Tuberc Lung Dis.

[B25] Marquis GS, Ventura G, Gilman RH, Porras E, Miranda E, Carbajal L, Pentafiel M (1990). Fecal contamination of shanty town toddlers in households with non-corralled poultry, Lima, Peru. Am J Public Health.

[B26] Lanata CF, Black RE, Gilman RH, Lazo F, Del Aguila R (1991). Epidemiologic, clinical, and laboratory characteristics of acute vs. persistent diarrhea in periurban Lima, Peru. J Pediatr Gastroenterol Nutr.

[B27] Vidal MF, Gilman RH, Ungar BL, Verastegui MR, Benel AC, Marquis G, Penny M, Lanata C, Miranda E (1991). Detection of Giardia lamblia antigen in children living in a Peruvian periurban shantytown (Pueblo Joven). J Clin Microbiol.

[B28] Graves KL, Leigh BC (1995). The relationship of substance use to sexual activity among young adults in the United States. Fam Plann Perspect.

[B29] Sanchez JL, Sjogren MH, Callahan JD, Watts DM, Lucas C, Abdel-Hamid M, Constantine NT, Hyams KC, Hinostroza S, Figueroa-Barrios R (2000). Hepatitis C in Peru: risk factors for infection, potential iatrogenic transmission, and genotype distribution. Am J Trop Med Hyg.

[B30] Slutske WS (2005). Alcohol use disorders among US college students and their non-college-attending peers. Archives of General Psychiatry.

[B31] Diaz T, Chu SY, Buehler JW, Boyd D, Checko PJ, Conti L, Davidson AJ, Hermann P, Herr M, Levy A (1994). Socioeconomic differences among people with AIDS: results from a Multistate Surveillance Project. Am J Prev Med.

[B32] Solorio MR, Asch SM, Globe D, Cunningham WE (2002). The association of access to medical care with regular source of care and sociodemographic characteristics in patients with HIV and tuberculosis. J Natl Med Assoc.

[B33] Fenton KA (2001). Strategies for improving sexual health in ethnic minorities. Curr Opin Infect Dis.

[B34] Brodbeck J, Matter M, Moggi F (2006). Association between cannabis use and sexual risk behavior among young heterosexual adults. AIDS Behav.

[B35] Fishbein M, Pequegnat W (2000). Evaluating AIDS prevention interventions using behavioral and biological outcome measures. Sex Transm Dis.

[B36] INEI (2007). Censos Nacionales 2007: XI Poblacion y VI de Vivienda. Lima, Peru.

[B37] Galvez-Buccollini JA, Paz-Soldan V, Herrera P, Delea S, Gilman RH, Anthony JC (2008). Links between sex-related expectations about alcohol, heavy episodic drinking and sexual risk among young men in a shantytown in lima, peru. Int Fam Plan Perspect.

[B38] Hendershot CS, Stoner SA, George WH, Norris J (2007). Alcohol use, expectancies, and sexual sensation seeking as correlates of HIV risk behavior in heterosexual young adults. Psychol Addict Behav.

[B39] Compton WM, Thomas YF, Stinson FS, Grant BF (2007). Prevalence, correlates, disability, and comorbidity of DSM-IV drug abuse and dependence in the United States: results from the national epidemiologic survey on alcohol and related conditions. Arch Gen Psychiatry.

[B40] Crosby R, Salazar LF, DiClemente RJ, Yarber WL, Caliendo AM, Staples-Horne M (2007). Condom misuse among adjudicated girls: associations with laboratory-confirmed chlamydia and gonorrhea. J Pediatr Adolesc Gynecol.

[B41] Hernandez LS, Winch PJ, Parker K, Gilman RH (2006). Understandings of reproductive tract infections in a peri-urban pueblo joven in Lima, Peru. BMC Womens Health.

[B42] Galanti GA (2003). The Hispanic family and male-female relationships: an overview. J Transcult Nurs.

